# PhyloGene server for identification and visualization of co-evolving proteins using normalized phylogenetic profiles

**DOI:** 10.1093/nar/gkv452

**Published:** 2015-05-09

**Authors:** Ilyas R. Sadreyev, Fei Ji, Emiliano Cohen, Gary Ruvkun, Yuval Tabach

**Affiliations:** 1 Department of Molecular Biology, Massachusetts General Hospital, Boston, MA, USA; 2 Department of Genetics, Harvard Medical School, Boston, Boston, MA, USA; 3 Department of Developmental Biology and Cancer Research, The Institute For Medical Research-Israel-Canada, The Hebrew University-Hadassah Medical School, Jerusalem, Israel

## Abstract

Proteins that function in the same pathways, protein complexes or the same environmental conditions can show similar patterns of sequence conservation across phylogenetic clades. In species that no longer require a specific protein complex or pathway, these proteins, as a group, tend to be lost or diverge. Analysis of the similarity in patterns of sequence conservation across a large set of eukaryotes can predict functional associations between different proteins, identify new pathway members and reveal the function of previously uncharacterized proteins. We used normalized phylogenetic profiling to predict protein function and identify new pathway members and disease genes. The phylogenetic profiles of tens of thousands conserved proteins in the human, mouse, *Caenorhabditis elegans* and *Drosophila* genomes can be queried on our new web server, PhyloGene. PhyloGene provides intuitive and user-friendly platform to query the patterns of conservation across 86 animal, fungal, plant and protist genomes. A protein query can be submitted either by selecting the name from whole-genome protein sets of the intensively studied species or by entering a protein sequence. The graphic output shows the profile of sequence conservation for the query and the most similar phylogenetic profiles for the proteins in the genome of choice. The user can also download this output in numerical form.

## INTRODUCTION

Phylogenetic profiling of a protein specifies the relative sequence conservation or divergence among orthologous proteins across a set of reference genomes. Proteins that function together, for example members of the same pathway or protein complex, often show similar pattern of conservation across phylogenetic clades. As an extreme case, when a species is no longer under evolutionary pressure to maintain a protein complex, pathway, organelle or specific function, the corresponding proteins are lost or show a strong divergence. Phylogenetic profiling based on the binary calls of the presence or absence of orthologs in the surveyed genomes produced impressive results in evolutionary analyses of a wide variety of prokaryotes and of eukaryotic organelles, such as the cilia (1,2) and mitochondria (3,4). When applied to eukaryotic genomes, however, this approach has been less effective (5–8), in part due to the difference in evolutionary rates in prokaryotes compared to eukaryotes: evolutionary rates and the resulting sequence divergence are higher in prokaryotes, due to various factors including shorter generation times and horizontal gene transfer. Although the binary phylogenetic profile can provide sufficient resolution for the analysis of divergence among prokaryotic proteins, it can be less accurate in eukaryotes. In eukaryotes, studying loss or retention of proteins without considering the levels of protein conservation between species might lead to higher amounts of noise in the data and limit the analysis. Quantitative estimates of orthologous sequence conservation may provide better resolution within the scale of evolutionary distances between eukaryotic species. At this scale, a simple presence of an orthologous protein in a genome may be insufficient to make conclusions about divergent evolution, and quantitative measures of sequence similarity would be more informative. These measures, however, should take into account evolutionary distances between compared species. For example, when comparing a human protein to its mouse and yeast orthologues, the sequence identity of 50% suggests a fast sequence divergence in mouse but relatively high conservation in yeast.

Recently, we developed Normalized Phylogenetic Profile (NPP) analysis based on a continuous measure of sequence similarity that is adjusted to evolutionary distance between species. The level of protein conservation for the ortholog in a given genome is normalized to the scale of conservation for all proteins in this genome, in effect taking into account the genome-wide distribution of conservation values expected for this individual species. This method captures protein sequence divergence and partial loss in the context of inter-species phylogenetic distances. Starting with query proteins from a few well-studied genomes, we surveyed and compared their phylogenetic profiles across full protein sets from 86 eukaryotic genomes. These analyses revealed novel components in various biological pathways including RNAi, m6A RNA methylation and the MITF pathway, as well as identified novel proteins associated with melanoma and other diseases (9–11).

Here we describe the implementation of the NPP algorithm on a web-based server, PhyloGene, which allows the user to submit protein queries, inspect the output in an interactive graphic format and download the output in numerical format.

## MATERIALS AND METHODS

To generate phylogenetic profiles, NPP (9–11) performs four steps: (i) for each protein from the genome of interest, run BLASTP (12) against the full protein sets of multiple eukaryotic genomes and choose the top BLAST hit for each genome; (ii) filter out low scores (BLAST similarity score <50) and query proteins that do not have homologs across a portion of genomes; (iii) normalize BLASTP bit scores by the score of the query to itself (i.e. top BLAST score against the same genome); (iv) calculate Z-scores on the population of normalized scores for each separate genome (species). Finally, Pearson correlation coefficients *r* are calculated between Z-score profiles for proteins of the query genome, and the top most similar phylogenetic profiles are recorded for each query protein. To estimate statistical significance of the resulting correlation, we use a naive null model of randomly shuffled species (columns of the table). For each pair of the compared profiles, we perform *N* = 1000 instances of random shuffling of values in one of the profiles and generate the corresponding random Pearson correlation coefficients (*r*). Based on the distribution of these random coefficients, we calculate a measure of statistical significance as the Z-score for the actual coefficient *r*.

The pre-computed top profiles along with the corresponding Pearson correlation coefficients and estimates of their statistical significance are displayed when the query protein is selected from the standard protein list. As a general rule of thumb, we observe that marginal Pearson correlation coefficient of 0.5 typically corresponds to a Z-score of ∼5.0, whereas highly significant Pearson correlation coefficient of 0.95 typically corresponds to a Z-score of ∼8.0. These Z-score values can be used as approximate cutoffs for insignificant and extremely significant similarities, respectively. It is important to mention that the Z-scores can be used only as rough estimation of the biological significance. A complimentary approach should take into account the biological knowledge about the query gene and estimate the significance of its co-evolved genes by the number of genes in the list that are known to interact with it. For example by integrating the PhyloGene results with other data sources like protein-protein interaction maps, results from high throughput screening or validated genetic pathways and find overlap between the lists as we previously showed (9–11).

When an individual query protein is submitted as a sequence, the workflow is modified: (i) run BLASTP with the submitted sequence as a query against protein databases of multiple eukaryotic genomes and choose the top BLAST hit for each genome; (ii) filter out low scores (BLAST similarity score <50); (iii) normalize BLASTP bit scores by the highest score among genome hits; (iv) transform top bit score against each ‘subject’ genome into a Z-score based on pre-computed mean and standard deviation of scores generated by BLAST searches with all proteins from the selected query genome against this ‘subject’ genome. Finally, Pearson correlation coefficients are calculated between the query Z-score profile and the pre-computed Z-score profiles for proteins from the genome of interest, followed by the selection of the top phylogenetic profiles with the highest similarity to that of the query protein. Pearson correlation coefficients for each of the top profiles and their statistical significance are calculated and displayed as described above.

PhyloGene is an intuitive and easy to use web tool that implements both of these modes. At the front page, the user can submit the query in the left panel and view the graphic output as a heatmap of sequence similarity patterns across multiple genomes in the right panel. The numerical output for more detailed analysis can be downloaded using a link to the Excel table in the left pane. The query can be submitted by (i) selecting a protein name from the menu of all proteins for a genome of the user's choice or (ii) submitting a protein sequence by pasting in a window or uploading the sequence file. In the left pane, the user can select the query genome of interest and the number of top most similar phylogenetic profiles to display. The front page also includes the link to a brief tutorial on using the tool.

### Input

The user can submit the query in the left pane of the front page by setting (i) organism of interest (*Homo sapiens, Mus musculus, Drosophila melanogaster* or *Caenorhabditis elegans*); (ii) query protein, by either selecting the gene of interest from the list or entering a protein sequence and (iii) the number of most similar phylogenetic profiles to display (the choice of 50, 100, 150 or 200).

### Output

**Figure 1.**
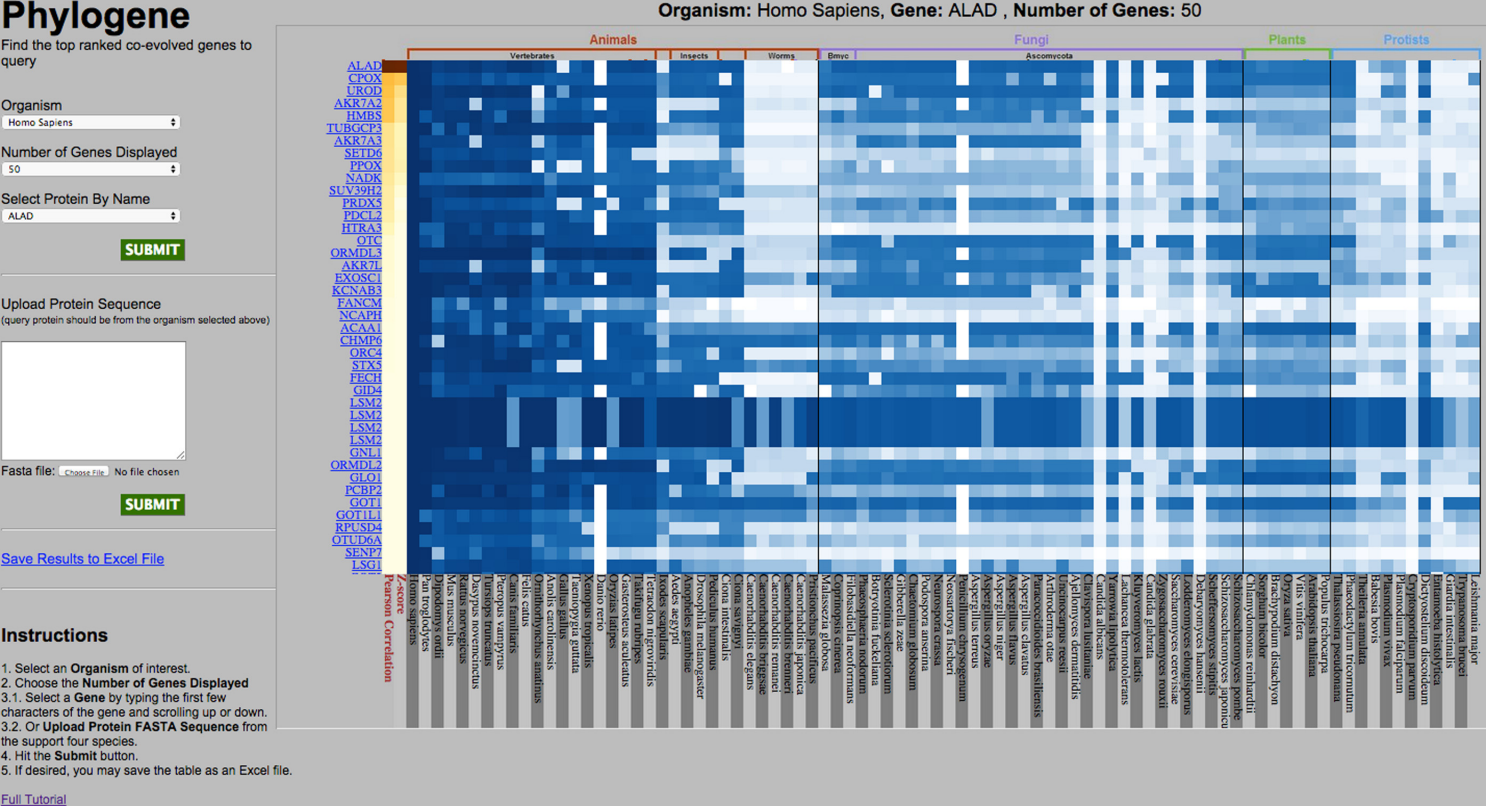
Front page of the PhyloGene server. In the left pane, the user can submit the query and set up basic parameters. After the query is submitted, the output is shown in the right pane as a heatmap, with rows corresponding to phylogenetic profiles with highest similarity to the profile of the query (shown as the top row). In this example, human ALAD protein was used as a query to detect top 50 human proteins with the most similar phylogenic profiles. Hovering the cursor over an element of the heatmap brings up the information about this element: human protein (also shown on the left of the heatmap), genome where the closest protein homolog was analyzed (also shown on the bottom of the heatmap) and the similarity value between the human protein and its homolog in this genome. Two leftmost columns, with elements colored in yellow–red hue, represent Pearson correlation coefficient and statistical significance (Z-score) of the similarity to the query.

The graphic representation of the results is displayed as a heatmap in the right panel of the page (Figure 1). The top of this panel shows the name of selected organism of interest, query protein and the required number of top similar phylogenetic profiles to display. The x-axis of the heatmap corresponds to the surveyed eukaryotic genomes, which are grouped into major taxa (Animals, Fungi, Plant and Protists) and subtaxa. These groupings are indicated on top of the heatmap, with additional vertical lines separating different taxonomic groups along the whole heatmap, for easier manual inspection of conservation patterns. The species names of the surveyed genomes are indicated at the bottom of the heatmap.

The y-axis of the heatmap corresponds to the proteins from the query genome whose phylogenetic profiles are most similar to the profile of the query protein. These profiles are shown as rows of the heatmap; the number of displayed profiles is selected by the user during submission process. The top row corresponds to the profile of the query protein. Protein names are displayed at the left edge of the heatmap. Hovering over a protein name with the mouse brings up a short description of the gene; clicking on a gene name opens the link to the Ensembl (http://www.ensembl.org) website with the information about this gene.

The heatmap displays the values of sequence conservation measure as color-coded squares ranging from white (no sequence similarity to the protein indicated to the left of the given heatmap row) to dark blue (very high sequence similarity). Hovering over a square brings up the protein name corresponding to this row, the genome in which the homology search was performed, and the sequence similarity value ranging from 0 to 1; this is also the value that the color of the square is based on.

The first two columns of the heatmap are colored in yellow–dark red hue and represent Pearson correlation coefficient and statistical significance of the similarity to the query. Hovering over these columns brings up the values of *r* and Z-score.

**Figure 2.**
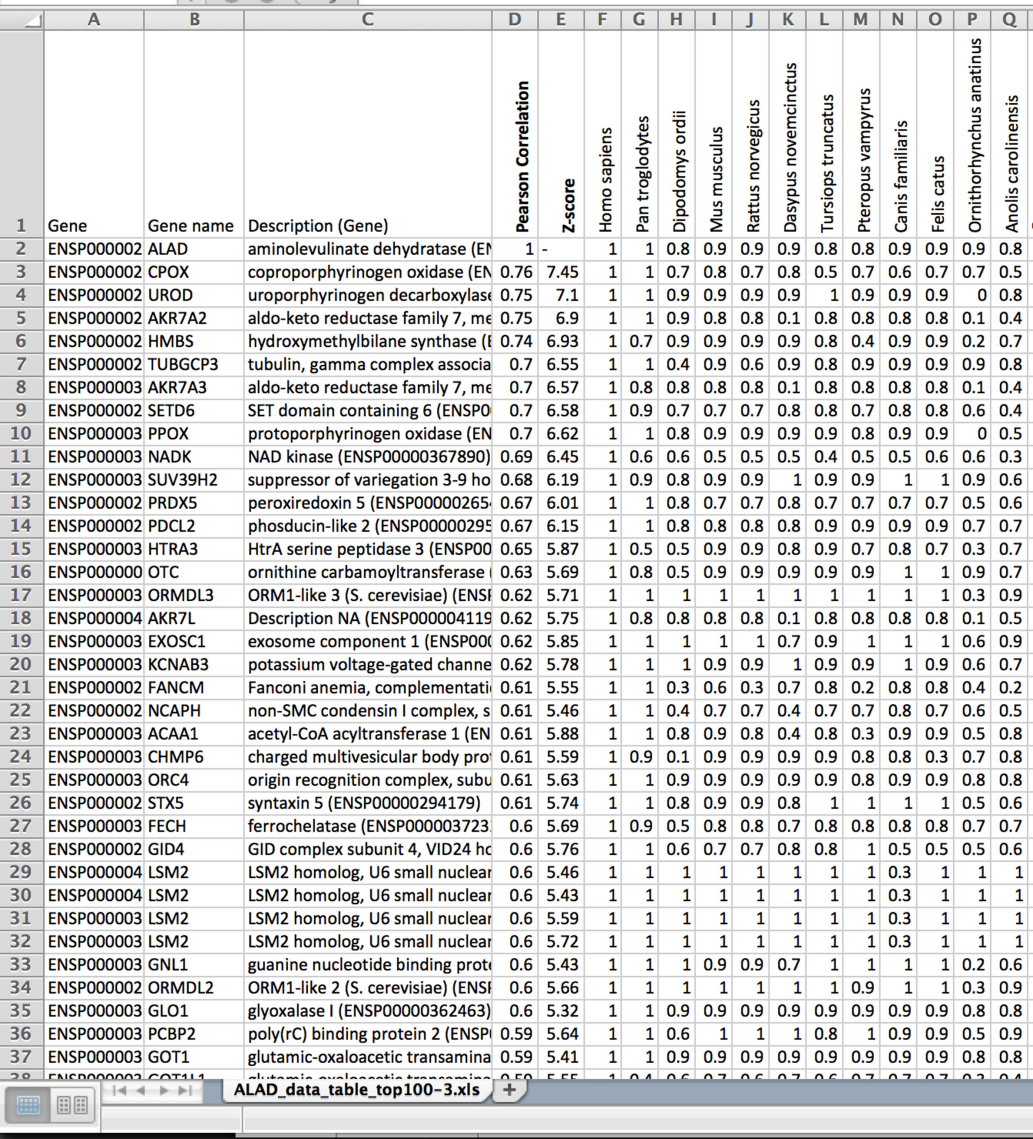
PhyloGene output as a numerical table. The table includes Ensembl IDs, names and short descriptions of proteins with highest profile similarity to the query, and the profiles themselves, as rows of highest protein similarity values across 86 species.

**Figure 3.**
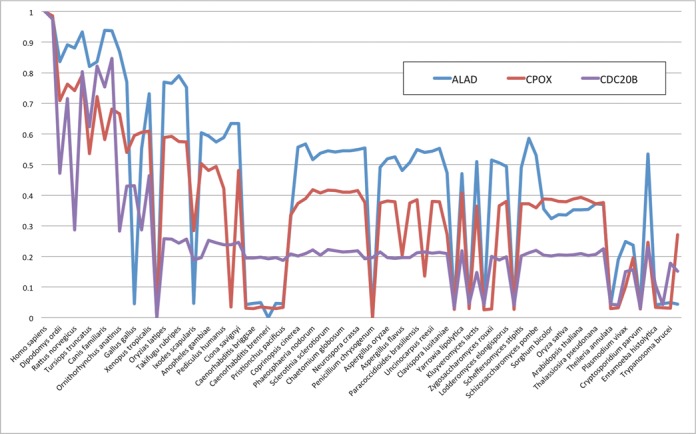
Example of more detailed analysis based on numerical output of PhyloGene. The phylogenic profiles of ALAD (blue), CPOX (red), the profile with the highest similarity ALAD, and CDC20B (purple), the 51^rd^ most similar protein profiles, were plotted as line graphs based on the values in the output table. The x-axis is the species (genomes) and the y-axis is the relative conservation values of proteins in these genomes compared to the human proteins.

In addition to visual inspection of the heatmap, the user can download the output in a numerical format as an excel table (Figure 2).The table has the same general structure as the heatmap and contains the protein names, short protein descriptions and the similarity values, which can be used for a more in-depth quantitative analysis of the results and visualization of specific gene profiles in different ways (Figure 3).

### Example of detected similarities between phylogenetic profiles

As an example of potential biologically relevant protein associations revealed by PhyloGene, the search with human delta-aminolevulinate dehydratase (ALAD) as a query confirms previously known genes in this pathway and suggests new functional links of ALAD with other human proteins (Figure 1). ALAD is the enzyme that catalyzes the second step in the porphyrin and heme biosynthetic pathway. Recessive mutations in the ALAD gene cause a rare form of acute hepatic porphyria. The PhyloGene search for similar phylogenetic profiles (Figure 1) retrieves six proteins that also belong to the heme biogenesis pathway (HMBS, CPOX, UROD, FECH, PPOX, in addition to ALAD) with Z-score > 5 and *r* > 0.6, consistent with their known functional association with ALAD. Interestingly, many of the proteins with similar phylogenetic profiles have been implicated in distinct biological processes from heme biosynthesis, such as transaminase activity. For example SETD1A, SETD1B AADAT and SUV39H2 are implicated in lysine degradation; 16 other proteins with phylogenetic profiles similar to heme biosynthesis genes, such as FDXR, GPX1 and GOT2 are associated with the mitochondrion and several proteins are involved in lipid biosynthesis or processing, such as three ORM1-like proteins. These proteins regulate the biosynthesis of sphingolipids; and mutations in these proteins have been associated with the development of childhood asthma (13). The similarity of their phylogenetic profiles to that of ALAD suggests the intriguing possibility of functional connection between heme and sphingolipid biosynthesis. In sum, these results confirm known associations of ALAD protein and suggest new hypotheses about potential interplay of ALAD, and heme biosynthesis pathway as a whole, with other proteins and pathways, providing new candidate genes for experimental validation and analysis.

The PhyloGene web server provides online access to our method of normalized phylogenetic profiling and has several immediate biological applications: (i) analyzing the conservation pattern of a given protein across eukaryotic genomes; (ii) predicting function of previously uncharacterized proteins, based on the phylogenetic profile similarity to proteins with known function; (iii) suggesting groups of proteins that co-evolved together and identifying new pathway members, based on inferred co-evolution with the known members of the pathway and (iv) revealing organismal adaptions to the environment on the level of proteins or pathways, which may provide clues to protein function.

## AVAILABILITY

http://genetics.mgh.harvard.edu/phylogene/.
